# Agrast-6: Abridged VGG-Based Reflected Lightweight Architecture for Binary Segmentation of Depth Images Captured by Kinect

**DOI:** 10.3390/s22176354

**Published:** 2022-08-24

**Authors:** Karolis Ryselis, Tomas Blažauskas, Robertas Damaševičius, Rytis Maskeliūnas

**Affiliations:** Faculty of Informatics, Kaunas University of Technology, 44249 Kaunas, Lithuania

**Keywords:** depth images, convolutional neural network, binary segmentation

## Abstract

Binary object segmentation is a sub-area of semantic segmentation that could be used for a variety of applications. Semantic segmentation models could be applied to solve binary segmentation problems by introducing only two classes, but the models to solve this problem are more complex than actually required. This leads to very long training times, since there are usually tens of millions of parameters to learn in this category of convolutional neural networks (CNNs). This article introduces a novel abridged VGG-16 and SegNet-inspired reflected architecture adapted for binary segmentation tasks. The architecture has 27 times fewer parameters than SegNet but yields 86% segmentation cross-intersection accuracy and 93% binary accuracy. The proposed architecture is evaluated on a large dataset of depth images collected using the Kinect device, achieving an accuracy of 99.25% in human body shape segmentation and 87% in gender recognition tasks.

## 1. Introduction

The fundamental issue of semantic segmentation has lately gained attention in the disciplines of computer vision and machine learning [1]. One of the crucial phases in developing complicated robotic systems, such as autonomous cars/drones, human-friendly robots, robot-assisted surgery, and intelligent military systems, is to assign a different class label to each pixel of a picture [2]. Convolutional neural networks (CNN), which have been applied to semantic segmentation, have unquestionably contributed to the recent rise in interest in this subject. One of the core concerns of computer vision has long been understanding a scene at the semantic level, but we have only recently saw practical solutions to the issue [3]. Therefore, it should come as no surprise that prominent businesses in the sector are now urgently tackling this issue alongside academic organizations that study artificial intelligence.

Depth sensor data analysis has been on the rise lately. With the prevalence of low-cost depth sensors such as Microsoft Kinect, different researchers found that depth data could be applied for 3D face recognition [4], fall detection [5], evaluation of upper extremity characteristics [6], fitness applications [7,8], exercise coaching [9], industrial worker activity monitoring [10], robotic applications [11], obstacle detection for visually impaired [12], anthropometric measurements [13], posture recognition [14] or general body tracking [15]. The RGB (red, green, blue) + depth data are used even more often in applications such as image encryption [16], object detection [17] and especially semantic object segmentation [18,19,20]. However, RGB cameras for some applications introduce security risks as shown by Roesner et al. [21]. Depth sensors do not pose as many security threats. Single-class (binary) segmentation is also sometimes useful. It was applied in areas as different as cloud segmentation [22], medical images [23] or human body segmentation [24].

If it is known that a certain object is already in the scene, the only issue to solve is segmenting the object from the background. This could be completed by existing semantic segmentation systems; however, they are more complex than actually needed, which means that they are also more difficult to train. The issues are acknowledged by Shazeer et al. [25], who suggest a solution to disable some parts of the network, or Huang et al. [26], who suggest a solution to train very large networks more efficiently. However, the training time for large neural networks still remains an issue, so smaller architectures are still required in order to reduce the time to deploy the CNN-based solution for some domains.

Our work has made the following significant contributions:

- An in-depth and organized examination of the most important deep learning models for semantic segmentation, their origins, and their contributions.
- A new convolutional deep learning model proposed for binary image segmentation.
- A comprehensive performance evaluation that collects quantitative metrics such as segmentation accuracy and execution time.
- A discussion of the aforementioned results as well as a list of potential future works that could set the course of future advances in semantic segmentation of depth images and a conclusion summarizing the field’s state of the art.

The organization of the remaining parts of the paper is as follows. Section 2 discusses previous deep learning models for semantic image segmentation. Section 3 introduces and describes the proposed neural network architectures. Section 4 presents the results of the experiments. Finally, Section 5 discusses the results and concludes.

## 2. Previous Work on Semantic Image Segmentation

VGG-16 is a neural network architecture introduced by Simonyan and Zisserman [27]. The authors suggested a fully convolutional neural network (FCN) that utilizes small 3 × 3 convolutional filters. The goal of VGG-16 is the classification of RGB images. The network consists of five segments of layers. Each segment consists of two or three convolutional layers with 3 × 3 filters with ReLU activation and a max pooling layer at the end. Each pooling layer reduces the dimensions of the previous layer by a factor of 2. The network uses a softmax layer to produce its output.

The VGG network itself was inspired by AlexNet [28]. The novelties that VGG introduced in comparison to AlexNet were small receptive filters (3 × 3 vs. AlexNet’s 11 × 11), which allowed a deeper network. AlexNet consists of 11 layers while VGG supports up to 19 layers. This property leads to better results. Research by Yu et al. [29] has shown that VGG-16 is better than AlexNet at removing background information. However, benchmarks performed by Canziani et al. [30] have shown that VGG and AlexNet carry a small amount of accuracy per parameter. This is a disadvantage of these models, as the researchers state that “VGG and AlexNet are clearly oversized”. VGG-16’s accuracy density was evaluated to be ~0.5% per million parameters, while that for AlexNet was 0.8% per million parameters. It was also shown by Paszke et al. [31] that smaller architectures are viable. They achieved 55.6% segmentation IoU accuracy, which is comparable to other state-of-the-art neural networks of the time. However, the authors state that their architecture requires 79 times fewer parameters to learn.

The VGG-16 network is popular and inspired many other architectures. SegNet is one of the networks based on VGG-16 [32]. It suggests an encoder–decoder architecture for semantic image segmentation—it takes RGB images as inputs and produces labels of semantic segmentation. VGG-16 acts as an encoder in this architecture. The decoder is the reverse of the encoder—it has the same layers but in reverse order, and max pooling layers are replaced with upsampling layers. The authors introduced the idea of using pooling indices computed in the max-pooling step in the encoder. The network was trained to segment objects on the road into 11 classes. SegNet was also applied in other areas of research such as brain tumor segmentation [33], detection of cracks in pavement [34] and semantic segmentation using event-based cameras [35]. This shows that SegNet is a very versatile architecture that can be applied in different areas. Mou et al. also suggest a VGG-16 encoder–decoder architecture based on VGG-16 with relation modules [36]. They include the spatial relation module and channel relation module, which are then aggregated. They help to identify long-term relations in the images.

A solution for binary image segmentation is SoftSeg [37]. The authors suggest that linear ReLU-based activation should be used instead of the sigmoid function in order to soften the boundary of the two classes.

TernausNet is another binary image segmentation network [38]. It is based on the so-called encoder–decoder (ED) architectures, which are divided into two halves and are often referred to as U-Nets in reference to the groundbreaking research by [39]. The spatial dimension is gradually decreased by the encoder using pooling layers, and the spatial dimension is gradually recovered by the decoder. By leveraging skip connections, each feature map in the decoder portion only obtains data directly from feature maps at the same level as the encoder part, enabling EDs to produce abstract hierarchical features with fine localization. The authors suggest using a VGG-11 based encoder–decoder with a fully connected layer replaced with a single convolutional layer of 512 channels. However, U-Net is criticized due to the blurring of extracted features and low-resolution information duplication [40].

He et al. [41] suggested a Spatial Pyramid Pooling Network that could produce a spatial pyramid representation of deep features independent of the input size (SPP-Net). The SPP-Net’s ability to feed CNNs with inputs of various sizes was its most significant contribution. Different-sized feature maps are always produced by feeding different-sized images into convolutional layers. However, the feature map produced by that layer would be fixed if a pooling layer, which comes before a decision layer, had stride values proportionate to the input size. The nature of this architecture prevents fine-tuning the layers before the SPP layer.

Recurrent Neural Networks (RNNs) and Convolutional Neural Networks (CNNs) are combined in the ReSeg model [42]. In order to more accurately localize the pixel labels, the input picture is fed through a CNN encoder that is similar to the VGG architecture and then processed by recurrent layers. Another related method is the DAG-RNN [43], which models long-range semantic relationships among picture units using a DAG-structured CNN+RNN network. To the best of our knowledge, semantic segmentation lacks solely recurrent structures, which is mostly because semantic segmentation necessitates an initial CNN-based feature encoding strategy.

Segmentation was also implemented by DeepLab v3 and Quick Shift combined via class voting by Zhang et al. [44]. Long et al. suggested a fully CNN [45] that does not have a full decoder and infers the output image directly from the bottleneck layer. However, all mentioned models, while useful and accurate, use RGB data. Since there are three channels, the input data are more complex than only the depth channel; therefore, the networks should also be possible to simplify for more efficient usage for depth image segmentation.

The ineffective loss of label localization within the feature hierarchy, the inability to process global context knowledge, and the lack of a method for multiscale processing may be summed up as the key shortcomings of FCNs. Therefore, the majority of the following research has focused primarily on offering new structures or methodologies as solutions to these problems.

The summary of this section is provided in Table 1.

## 3. Methodology

### 3.1. Workflow

Prediction using the Agrast-6 model is implemented in a Java project. The model is loaded from a disk from the Tensorflow saved model format; the image is converted to a format acceptable by Tensorflow Java library and passed to it. The result is then fetched and converted back from the Tensorflow data structures.

### 3.2. Suggested Neural Network—Agrast-6 Architecture

The suggested neural network model—Abridged VGG-Based Reflected Architecture for Segmentation Training-6 (Agrast-6)—is an abridged modification of SegNet [32]. The task solved by SegNet is semantic segmentation. In order to solve this task, SegNet architecture introduces over 32,000,000 trainable parameters. This architecture can be simplified for simpler tasks.

First, SegNet is based off VGG-16, which itself is a network of 16 layers [27]. However, the first three layers are fully convolutional layers which only work with 224 × 224 × 3 BGR images. Depth data only has one channel. This limitation could be overcome by repeating the same channel three times. However, this research does not intend to use pre-trained weights from SegNet or VGG models; therefore, it makes sense to simplify the model instead. These layers are dropped in Agrast-6.

Next, VGG-16 consists of blocks of layers. There are 5 blocks; each block consists of either 2 or 3 convolutional layers with a 3 × 3 filter and a max pooling layer at the end. Each max pooling layer reduces both dimensions of the image by 2. The depth of convolutional layers is increased with each block, starting with the depth of 32 at the first layer up to 512 at the last layer. This gives extra dimensions for the network to learn the features. However, in binary image segmentation, there are fewer features to learn, and the model is more simple than in semantic segmentation. Thus, Agrast-6 leaves 3 blocks of layers instead of 5, and each block only has one convolutional and one max pooling layer. The depth of convolutional layers is also decreased to 32, 128 and 256, while max pooling is more aggressive with the first two layers reducing image dimensions by a factor of 4 and the last layer reducing image dimensions by two. In that case, the total dimensional compression is the same as in VGG-16, but it is completed quicker with more aggressive max pooling layers and fewer convolutional layers.

The first part acts as an encoder. The next part, introduced in SegNet, is the decoder. It tries to generate a new segmentation image from the encoded features. The decoder is a reflection of the encoder with max pooling layers replaced with upsampling layers. Changes in dimensionality are kept the same in both the encoder and the decoder. This idea is also used in Agrast-6. Three blocks of upsampling convolutional layers are used to generate the image from the encoded features. However, in the case of SegNet, the depth dimension of the last layer is 32, because it has to learn many labels. In binary segmentation, there are only two possible layers, so there is no reason to have 32 outputs per pixel. Thus, one more convolutional layer of depth dimension reduction is introduced.

**Figure 1:**
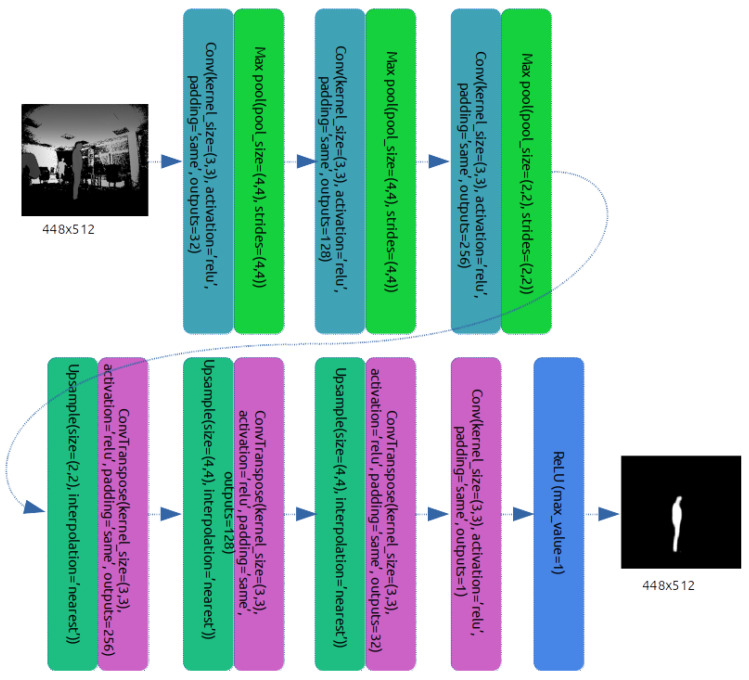
Architecture of the proposed Agrast-6 deep learning model for binary segmentation tasks.

Originally, SegNet uses the Softmax activation function as its last layer. Softmax tends to yield better results with multi-class classification tasks [46]. However, in case of a binary classification, ReLU was observed to perform better. This was shown in the works of Agarwal et al. [47]. As a result, ReLU activation is used in the Agrast-6 network as the activation of the last layer. To sum up, the full architecture of Agrast-6 is 6 layers of encoders (3 convolution–max pooling pairs), 6 layers of decoders (3 upsampling–convolution pairs), a feature reduction convolutional layer and a ReLU classifier. The full architecture is shown in Figure 1. The notation used in the figure is based on the Tensorflow terminology. The padding value of “same” for convolutional and transposed convolutional layers means that the convolution is applied to all pixels and edge pixels are zero-padded. ReLU activation at the end has a max value set to one. This option limits the final value of this layer to 1, i.e., ReLU is applied, and if the value coming from this layer is greater than 1, it will be set to 1; otherwise, the original ReLU function output will be used.

### 3.3. Neural Network Implementation

**Figure 2:**
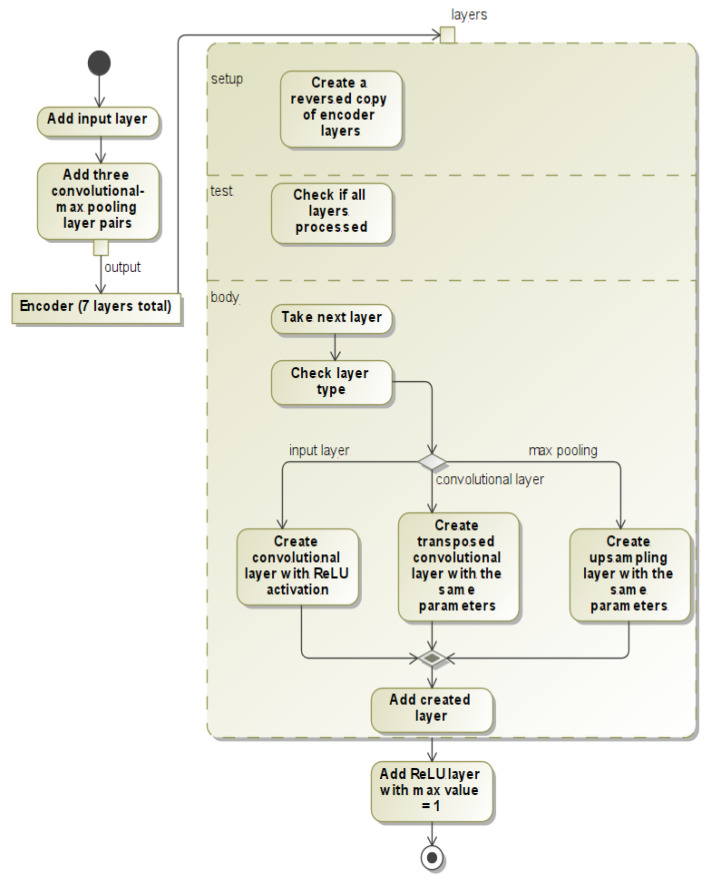
UML activity diagram of neural network creation process.

The decoder is automatically generated from the encoder. The decoder generator iterates over the encoder layers in reverse and produces new layers. The upsampling layer is generated if a max pooling layer is found; the deconvolutional layer is generated if a convolutional layer is found; the ReLU layer is generated if an input layer is found. This process is shown as a UML activity diagram in Figure 2. It outputs the network shown in Figure 1 and guarantees that the architecture is mirrored. Copied parameters for convolutional–transposed convolutional layers are filters, kernel size, activation and padding. The upsampling layers are generated by setting the size of the layer as encoder strides. The final transposed convolutional layer has 1 filter, a 3 × 3 kernel size and ReLU activation.

## 4. Neural Network Training and Evaluation

### 4.1. Dataset

The Agrast-6 neural network has been trained using the dataset of 220,000 images. The dataset is self-collected depth image sequences (videos) captured using a Kinect 2 camera. The images contain people sitting, standing or doing different poses, for example, standing on one leg or raising their hand. The dataset is captured indoors. A total of 45 people participated in data capture as actors. It is split into two parts: simple poses (standing and sitting on the chair, open environment) and complex poses (different poses from raised hands to crouch to laying on the ground, more cluttered environment). Ground truth binary masks have been obtained semi-automatically with human supervision using specialized software. The size of the dataset is 220,000 depth images with their respective foreground masks.

Kinect depth image dimensions are 424 × 512; however, the Agrast-6 network downscales the image in max pooling layers and then upscales it in upsampling layers by a factor of 32. Since 424 does not divide by 32, the data were right-padded with values of 0 both in the depth frames and ground truth frames.

### 4.2. Settings

Agrast-6 is implemented using Python programming language and a Tensorflow 2 library. Tensorflow includes the model of VGG-16, which was modified to produce the encoder. The training was performed on a system with AMD Ryzen 3900X CPU and NVidia 1660 SUPER GPU. The model has 1,200,000 trainable parameters.

### 4.3. Training

The whole dataset is divided into train and test parts, the train part is 80% of the data, and 20% are used for testing.

The learning rate has been set to 0.0001 with Adam optimizer. However, after 100 batches, the human shape is already visible, while after 6400 batches, the human is clearly visible. After 16,200 batches of data (just over 1/3 of the first epoch), the human silhouette is already bright, which shows that the confidence of the network has become high.

A batch of this size consists of about 4900 frames, which means that 8.2 GB of memory is used for one batch of images. The frames from all images are put into a single collection which is then shuffled and fed into the training method. When all frames are exhausted, the next 20 image sequences are loaded, and the process repeats until all data are exhausted. The same is completed for test images, but the batch size is reduced to 5. This process is repeated for each training epoch. It takes about 5 h to train one epoch and 1 h to validate the model against the test dataset.

Table 2 summarizes the hyperparameters used during training. The convolutional and deconvolutional layers all had the same kernel size and ReLU activation. The max-pooling window size was the same as dimensionality reduction. Adam optimizer with a learning rate of 0.0001 was used, and it optimized for a binary cross-entropy function.

### 4.4. Quantitative and Qualitative Validation Analysis

The neural network was evaluated using the standard training metrics. In the first four epochs, it has shown significant improvements in loss, precision and recall values with both test and train data. The next epochs have shown little improvement. After five epochs of training, the network peaked at its train precision and accuracy, while metrics with the test dataset continued to improve. Learning progress details are shown in Table 3.

**Figure 3:**
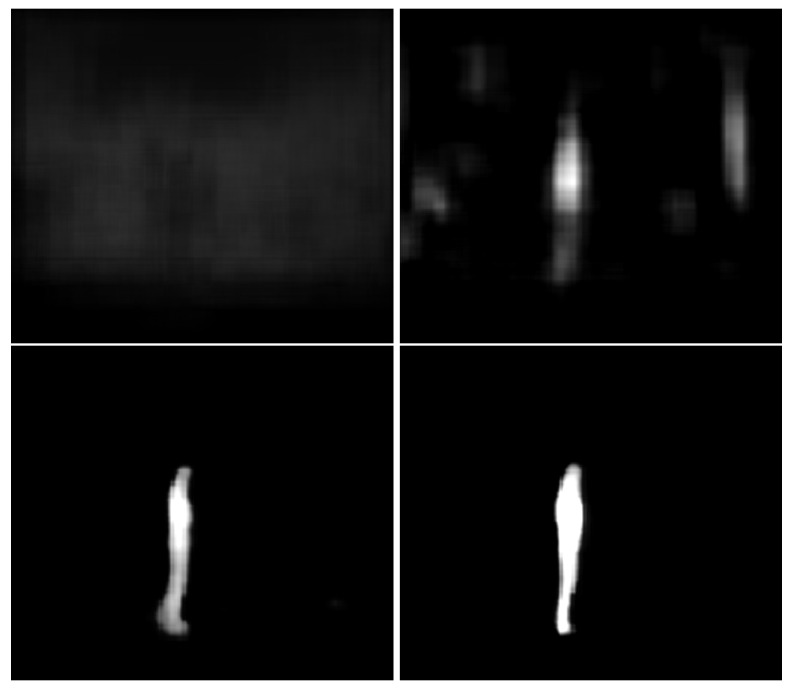
Neural network output after training batches 0, 100, 8400, and 16,200.

The results suggest that the neural network has not overfit the data, because all test metrics are high and started decreasing. Another interesting property of the training is the early stages of learning. Figure 3 shows how the network immediately started learning toward the correct output. Batch zero is not entirely random; however, not much is visible yet.

**Figure 4:**
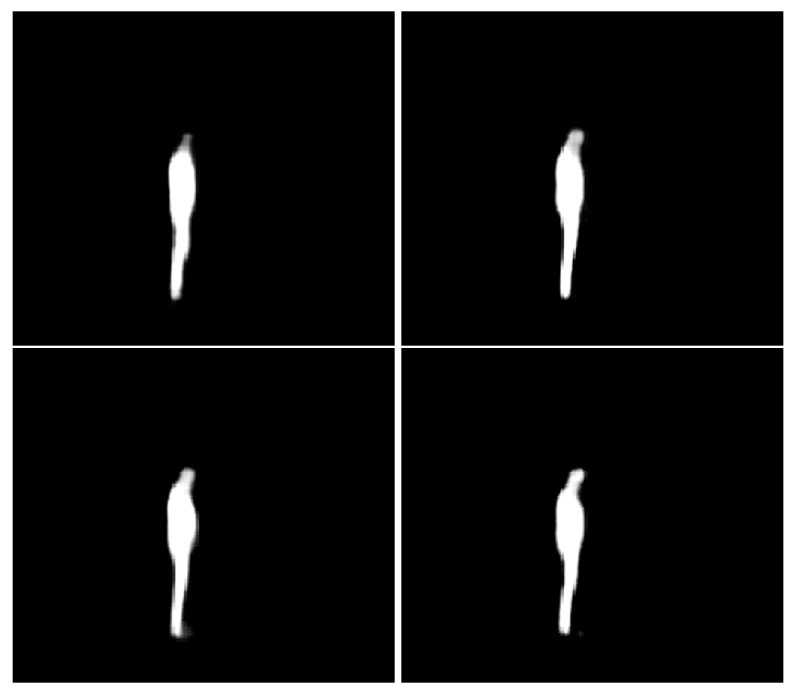
Neural network output after epochs 2, 5, 6 and 9.

Figure 4 shows the outputs of the network after different epochs. The network is not quite sure about the head after two epochs; however, it grows with each successive epoch. After seven epochs, the network seems to have learned about the head, which is a relatively small part of the body with unique features and contributes less to the loss function. After nine epochs, the output is bright white, which shows the high confidence of the network.

Both Figure 3 and Figure 4 show outputs for an image from the test set which has not been used to train the network. This qualitative output evolution suggests that the network is learning new features with each epoch.

### 4.5. Segmentation Evaluation via Cross-Intersection Accuracy and mIoU

The Agrast-6 network with weights learnt in nine epochs was loaded into a Java custom benchmark tool to test the mIoU and cross-intersection accuracy metrics. The latter was calculated as

(1) $$a=\frac{n(A\cap G)}{n(A)}\times \frac{n(A\cap G)}{n(G)}$$

where *A* is a set of points marked by Agrast-6, and *G* is a set of ground truth points. An average of accuracy values has been computed for each frame sequence. Pixels with over 0.5 confidence were included in the segmentation output.

**Figure 5:**
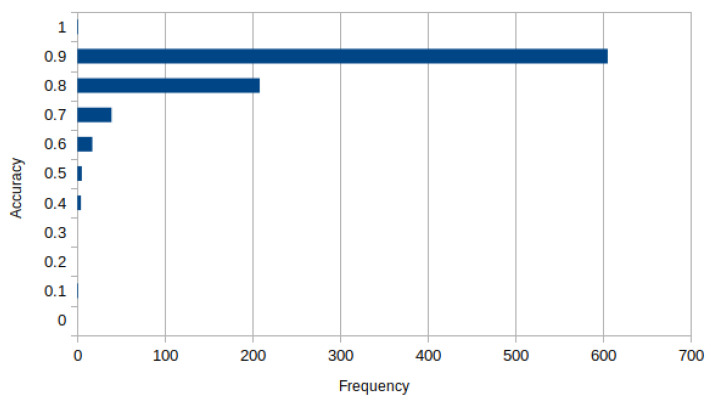
A histogram of segmentation accuracy value distribution.

It was found that the average score of cross-set intersection is 85.7% while mIoU is 86.5%. However, most of the per-sequence cross-set intersection accuracy values (69%) fall into the 90–100% accuracy category. In addition, 7.5% of the frame sequences were processed at the accuracy of less than 80%. A detailed distribution of accuracy values is shown in Figure 5.

**Figure 6:**
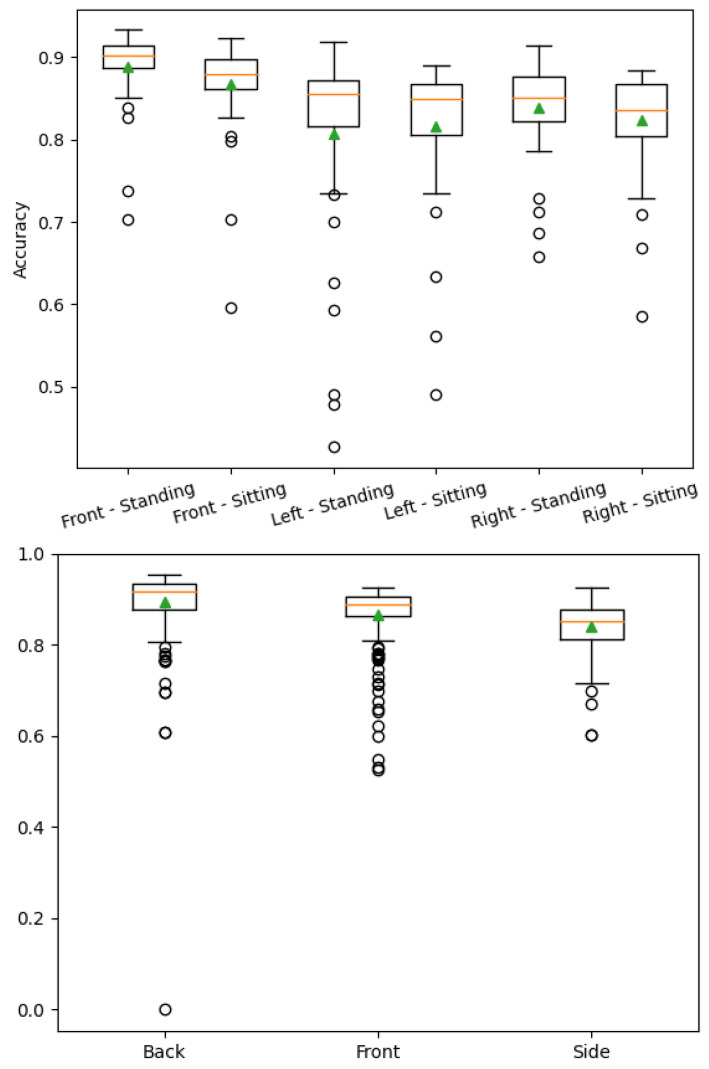
Accuracy value analysis by dataset. Left—standing and sitting people, right—people in complex positions.

Figure 6 shows accuracy values dissected by dataset and camera angle. The network is more stable with a more complex dataset; however, the accuracy is similar in both datasets. Side views of the human seem to be more challenging for Agrast-6 to segment. This may be due to the smaller surface area visible by the camera. Back and front views were easier to segment due to a larger visible surface area of the body.

**Figure 7:**
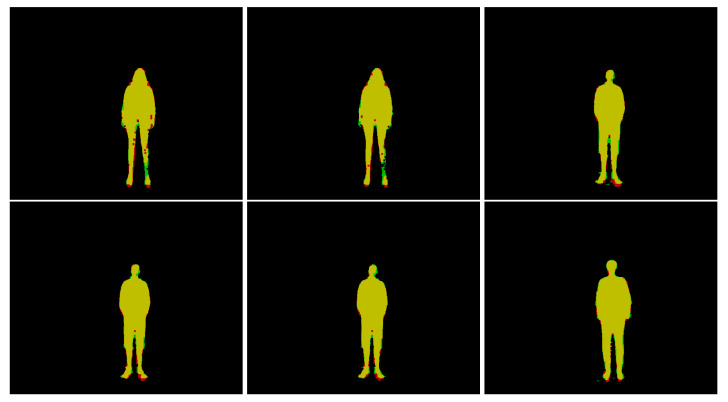
Examples of most typical segmentation accuracy images. Accuracies of 86%, 88%, 89%, 90%, 91% and 92%, respectively.

Figure 7 shows example segmentations with the most prevalent accuracy values. The green color represents false-negative pixels, while red represents false positive, and yellow represents true positive. (*A* is red, *G* is green, intersection is yellow). It is qualitatively visible that the leg is the hardest part of the body to process for the neural network. However, it is worth noting that ground truth is also the most difficult for humans to acquire, so these two issues may be related.

### 4.6. Gender-Wise Accuracy Comparison

The dataset used for training and evaluating the Agrast-6 model consists of depth images of people with balanced proportions of male and female participants. The research conducted by Karastergiou et al. has shown that body fat is distributed in different patterns in male and female bodies [48]. This means that gender impacts the shape of the body, which could lead to different accuracy of the segmenter for different genders. Therefore, it was decided to evaluate how the network performs for different genders.

The results have shown that females are detected with more accuracy in both sub-datasets. Detailed results are outlined in Table 4. Since the results are consistent across both datasets, females could be seen as more easily segmentable by AGRAST-6 architecture. Note that there are more male samples in the complex dataset; however, female silhouettes were easier to learn.

The observed results may be caused by the clothing differences between male and female participants. For example, some female subjects were wearing a dress during the capture, which may be easier for the neural network to localize. On the other hand, some male participants were wearing black jeans that cause a lot of noise around the leg area and makes it difficult for the network to segment correctly. Female participants also tend to have longer hair (there were zero male participants with long hair), and this may have helped to correctly segment the head, which, as shown in Figure 4, was the most difficult part of the body to learn.

### 4.7. Qualitative Error Analysis

**Figure 8:**
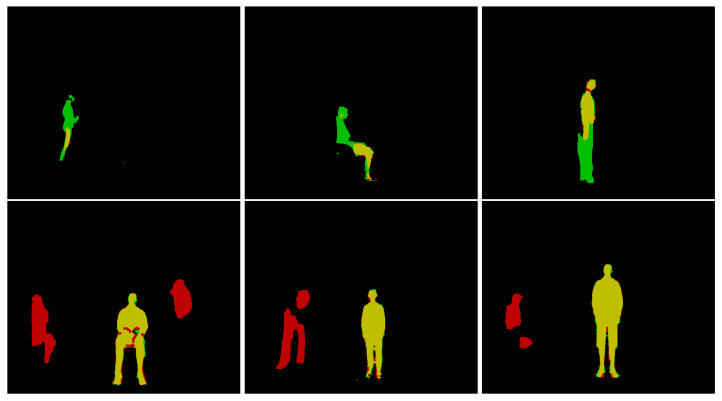
Examples of low-accuracy segmentation outputs.

**Figure 9:**
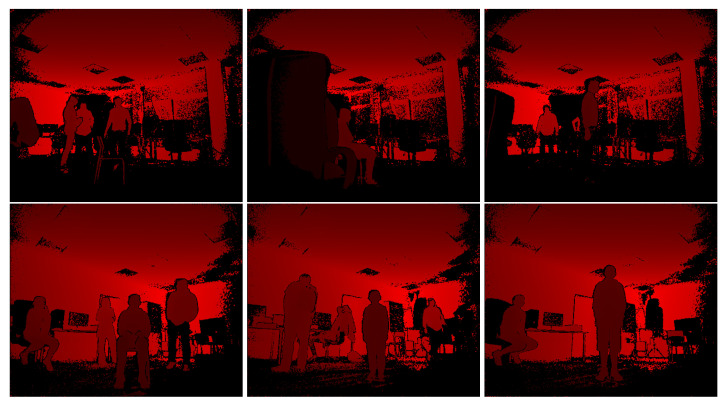
Examples of depth as seen by the camera in images in Figure 8.

Some examples of errors made by Agrast-6 are provided in Figure 8. The first two images show examples where the network almost completely failed to segment the depth image. Only the thigh was included in the output for the first image (yellow color shows the correctly segmented part of the body), and the legs were included in the second example. The person in the second image was partially occluded by another object, which may have caused troubles in segmenting the output. The third image is an example with the black jeans—the whole bottom part of the body was not included in the segmentation output, which shows that the network has not yet learnt this type of noise in “Kinect” depth images. The bottom row shows images where the person was segmented correctly or almost correctly, but extra artifacts were included in the segmentation output (shown in red). These objects have shapes somewhat similar to the human body and were mistakenly included as a human body. However, the first five images are from the bottom 1% of the images by accuracy while the last one is in the bottom 7%. Therefore, these mistakes are made only in some cases. Figure 9 showcases depth as seen by the camera for these images.

### 4.8. Prediction Performance

The computational performance of the Agrast-6 model is summarized in Table 5. A performance benchmark has been run on a system with AMD Ryzen-9 3900X CPU (469 GFlops) and NVidia GTX 1660 SUPER GPU (5.03 TFlops). Tensorflow for Java has been used to perform the benchmark, and GPU utilization was enabled. Measurements include float buffer (input) initialization, prediction session run and result fetch to a new float buffer. Very little variation was observed between the runtime, which was expected since AGRAST-6 accepts images of the same dimensions. The only exception was the first frame, which took longer than the others. The average prediction time was 166 ms, the shortest time was 154 ms, and the longest (first frame) was 229 ms. A standard deviation of 12.8 was observed, which shows that the processing time is quite stable.

### 4.9. Comparison with Other Solutions

Palmero et al. suggested a multi-model human body segmentation model from the RGB-Depth and thermal data [49]. The authors introduce several descriptors and fuse them together. They found that random forest classifier worked best to segment the images at 79% overlap. Zeppelzauer et al. constructed another random forest solution that segments depth data of rock art [50]. Their solution works by computing deviation maps, extracting valleys and peaks from these maps and classifying the data using a random forest classifier. They achieve an accuracy of 60% measured in the Dice similarity coefficient. Wang et al. used CNN to segment brain tumors from medical images [51]. They used three different neural networks to process different stages of segmentation. WNet [51] segments the tumor itself, which is the relevant part for this comparison. It consists of 14 blocks of convolutional layers each having one to three layers. The partial output is also captured and later combined. This architecture reached a Dice score of 91%. The author suggested a geometrical method to segment point cloud semi-automatically. It uses an expanding bounding box to segment the depth data represented by a point cloud [52]. This research used the same dataset. However, the bounding box approach, while being focused on speed, yielded highly varying results. For the simpler part of the dataset, it averaged at 76%.

This overview suggests that CNN-based models are currently among the best-performing techniques to solve segmentation problems, and the Agrast-6 architecture is no exception.

Table 6 shows a comparison of different neural network sizes. The networks from the older generation such as AlexNet or VGG-16 have a very large number of parameters, 62,000,000 for AlexNet and even 134,000,000 for VGG-16. Such large architectures are difficult to train due to the huge amount of data required and other problems such as exploding gradients. The newer generation of neural networks is much smaller. Both SegNet and U-Net networks have around 30,000,000 parameters. However, Agrast-6 shows that even these amounts of parameters are overkill for binary image segmentation. The proposed architecture has only 1,250,000 parameters. This leads to a much smaller model size; when exported using the Tensorflow saved model format, it takes only 15.4 MB of disk space, while SegNet, the next most lightweight model, takes 117 MB, which is 7.6 times larger than Agrast-6. Segmenting neural networks tends to work slower than classifying neural networks with respect to parameter count. This is evident from the inference time comparison shown in Table 6. AlexNet and VGG-16 inference times are lower than SegNet or U-Net inference times despite having more parameters to be trained. On the other hand, image resolution has a big impact on segmenting forward pass. SegNet and U-Net have a similar number of parameters; however, SegNet is two times faster, as the image input size was much smaller. As a result, Agrast-6 architecture’s forward pass takes 292 ms, which is faster than SegNet despite much higher resolution, and it produced a relatively high accuracy. This benchmark was performed using an Intel i5-8250U CPU (163 GFlops); all models used a Tensorflow implementation.

## 5. Discussion and Conclusions

This article presented an abridged VGG-based reflected architecture for segmentation training, which is named Agrast-6 (“Agrastas” means “gooseberry” in Lithuanian). It has six encoder layers, seven decoder layers and a ReLU activation layer, which reduces the number of trainable parameters by a factor of 27 in comparison with SegNet [32]. However, the problem of binary segmentation is less complex than semantic segmentation, which allows a simpler network, which, in turn, reduces the time required for training significantly. The simpler model did not cause low accuracy, as even after nine epochs of training, this model already achieves binary segmentation accuracy comparable to much heavier state-of-the-art models. The Agrast-6 architecture has been trained using a real-life indoor dataset collected using a Kinect camera, where one person is semantically an obvious foreground and has shown the average cross-intersection accuracy of 86% and mIoU of 87% at segmenting the data. This means a much higher accuracy per parameter (70% accuracy per 1,000,000 parameters), which is impossible to achieve with large architectures. This could be further increased using more training epochs, as test accuracy and precision metrics have still been increasing.

The accuracy of the network could further be improved by optimizing and fine-tuning the hyperparameters of the architecture—this is a proof-of-concept work that shows that this kind of architecture is viable for binary segmentation. However, it takes a lot of time and data to optimize the hyperparameters as the learning time of Agrast-6 is quite large, which will require a careful selection of the optimization heuristics. Unfortunately, it is not possible to reuse pre-trained weights of another model such as SegNet due to the different architecture and different domains of application. Another way to improve segmentation accuracy is to add a post-processing step, since the segmentation sometimes includes separate distinct objects.

## Figures and Tables

**Table 1:** Overview of previous work on semantic image segmentation.

Year	Model	Novelty	Major Drawback
2012	AlexNet [28]	Depth of the model	Ineffective and lower accuracy than later models
2014	VGG-16 [27]	Small receptive fields	Heavy model, computationally expensive
2015	U-Net [39]	Encoder–decoder architecture	Blurred features, slower due to decoder
2015	SPP-Net [41]	Variable image size adaptation	Cannot fine-tune convolutional layers before SPP layer
2015	FCNN [45]	Adaptation into fully convolutional networks	-
2016	ReSeg [42]	Recurrent layer	Features must be extracted using other techniques
2017	SegNet [32]	Decoder non-linear upsampling	Slower due to decoder
2021	SoftSeg [37]	Normalized ReLU activation and regression loss function	Hard to evaluate due to fuzzy boundaries

**Table 2:** The values of Agrast-6 model hyperparameters.

Hyperparameter	Value
Convolutional layer kernel size	3 × 3
Convolutional layer activation function	ReLU
Max-pooling pool size	4 × 4, 2 × 2 for last layer
Optimizer	Adam
Optimizer learning rate	0.0001
Loss function	Binary cross-entropy

**Table 3:** Training results of the proposed deep learning architecture. The best performance is indicated in bold.

Epoch	Train				Test			
	Loss	Precision	Accuracy	Recall	Loss	Precision	Accuracy	Recall
4	0.0161	0.9415	0.9939	0.9444	0.0430	0.9308	0.9916	0.9030
5	**0.0150**	**0.9457**	**0.9944**	**0.9490**	0.0347	**0.9317**	0.9920	0.9106
6	0.0158	0.9440	0.9940	0.9440	0.0329	0.9209	0.9919	0.9206
7	0.0152	0.9452	0.9942	0.9469	0.0351	0.9226	0.9915	0.9102
8	0.0164	0.9412	0.9938	0.9433	**0.0324**	0.9195	0.9921	**0.9265**
9	0.0162	0.9437	0.9939	0.9421	0.0325	0.9316	**0.9925**	0.9194

**Table 4:** Male vs. female detection accuracy.

Dataset	Gender	Mean Cross-Set Intersection	mIoU
Simple	male	82.2%	82.4%
Complex	male	86.7%	87.1%
Simple	female	85.3%	85.8%
Complex	female	87.3%	87.6%

**Table 5:** Performance of the Agrast-6 model.

Parameter	Value
Computational speed achieved on AMD Ryzen-9 3900X CPU	469 GFlops
Computational speed achieved on NVidia GTX 1660 SUPER GPU	5.03 TFlops
Average prediction time	166 ms
Shortest prediction time	154 ms
Longest prediction time	229 ms

**Table 6:** Comparison of segmentation methods.

Method	Accuracy	Input Type	Based on	Purpose	Parameters	Model File Size	Inference Time	Input Size
Multi-modal RF RGBD + T [49]	78%	RGBD + T	Random forest + descriptors	Segmentation	-	-	-	-
Rock depth + RF [50]	60%	Depth	Random forest + deviation maps	Depth segmentation	-	-	-	-
WNet [51]	91%	Medical depth	CNN	Segmentation	-	-	-	-
AlexNet [28]	60%	RGB	CNN	RGB classification	62 M	233 MB	52 ms	227 × 277
VGG-16 [27]	75%	RGB	CNN	RGB classification	134 M	528 MB	215 ms	224 × 224
SegNet [32]	60%	RGB	CNN	Semantic RGB segmentation	32 M	117 MB	341 ms	340 × 480
U-Net [39]	92%	RGB	CNN	RGB binary segmentation	30 M	386 MB	676 ms	512 × 512
Auto-expanding BB [52]	76%	Depth	Geometrical	Binary depth segmentation	-	-	60 ms	424 × 512
Agrast-6 (this paper)	86%	Depth	CNN	Binary depth segmentation	1.25 M	15.4 MB	292 ms	448 × 512

## Data Availability

The data presented in this study are available on request from the corresponding author. The data are not publicly available due to privacy requirements.
